# Impact of naturally occurring hemagglutinin substitutions on antigenicity and fitness of influenza A(H5N1) virus

**DOI:** 10.1038/s44298-025-00154-5

**Published:** 2025-10-02

**Authors:** Li Wang, Masato Hatta, Chenchen Feng, Paul Carney, Melvyn R. Almanzar-Jordan, Jieru Wang, Jaber Hossain, Ginger Atteberry, Nacyra Assad-Garcia, Sydney Sheffield, Juliana DaSilva, Juan A. De La Cruz, Yasuko Hatta, Monique C. Johnson, Julia Frederick, Rebecca Hart, Kristine A. Lacek, Malania Wilson, Lisa M. Keong, Xudong Lin, Ying Huang, Michael Currier, Gene Tan, Sanjay Vashee, Vivien G. Dugan, Benjamin Rambo-Martin, Han Di, Marie K. Kirby, C. Todd Davis, James Stevens, Bin Zhou

**Affiliations:** 1 Influenza Division, National Center for Immunization and Respiratory Diseases, Centers for Disease Control and Prevention, Atlanta, GA USA; 2 Division of Core Laboratory Services and Response, Office of Laboratory Systems and Response, Centers for Disease Control and Prevention, Atlanta, GA USA; 3 J. Craig Venter Institute, Rockville, MD USA; 4 J. Craig Venter Institute, La Jolla, CA USA; 5 University of California San Diego, La Jolla, CA USA

**Keywords:** Immunology, Microbiology

## Abstract

In 2024, a human infection with clade 2.3.4.4b highly pathogenic avian influenza A(H5N1) virus was identified in the United States in an individual with no known exposure. Genetic analysis revealed two hemagglutinin (HA) substitutions, P136S and A156T, which may alter viral antigenicity. Virus isolation was unsuccessful, preventing timely serologic analysis. To overcome this limitation, we generated recombinant viruses by reverse genetics and characterized the effects of the substitutions on antigenicity, receptor binding, and replicative fitness. The A156T substitution introduced a potential N-linked glycosylation site, resulting in altered antigenicity and reduced replication in primary human nasal epithelial cells and ferrets. Importantly, the A(H5N1) candidate vaccine virus (CVV) IDCDC-RG80A, which possesses HA-T156, remained antigenically effective against viruses with and without these substitutions. These findings highlight the importance of sequencing, reverse-genetics approaches, and antigenically similar CVVs such as IDCDC-RG80A, for pandemic preparedness against evolving clade 2.3.4.4b A(H5N1) viruses.

## Introduction

Highly pathogenic avian influenza (HPAI) A(H5) viruses of clade 2.3.4.4b have spread extensively through wild bird populations to diverse geographic regions including Asia, Africa, Europe, the Americas, and Antarctica^1^. The viruses have also been detected in numerous mammalian species such as domestic cats, lions, bears, foxes, seals, and skunks, among others^1,2^. On March 25, 2024, the first outbreak of clade 2.3.4.4b A(H5N1) virus in dairy cattle was reported in Texas. As of April 24, 2025, A(H5N1) viruses have been detected in more than 1,000 dairy herds in the United States. Additionally, 70 human cases have been identified in the U.S., most of which were exposed to infected or potentially infected dairy herds or poultry^3^. Although human infections with 2.3.4.4b viruses remain rare, the potential for these viruses to mutate and adapt to allow for transmission between humans and other mammals is a significant public health threat.

A human case of A(H5N1) virus detected in Missouri, with no known exposure to infected animals and carrying unique hemagglutinin (HA) changes P136S and A156T relative to the H5N1 viruses associated with the U.S. cattle outbreak, raised concerns about the potential impact of these substitutions on viral properties. The P136S substitution (H3 numbering: 140) is located in antigenic site A, adjacent to the receptor-binding domain 130-loop, while A156T (H3 numbering: 160) lies in antigenic site B and within the receptor-binding 150-loop based on mature H5 HA1 numbering. Notably, A156T also introduces a potential N-linked glycosylation site at residue 154. The 156 T residue has been found in several earlier H5 clades, and previous studies in H5 and other subtypes have linked it to altered antigenicity, receptor-binding properties, HA-NA balance, pathogenicity, and transmissibility, although reported effects vary across virus subtypes and clades^4–11^. Despite these prior reports, this is the first time these specific substitutions have been identified in a human case in the U.S., and their impact on antigenicity and fitness of clade 2.3.4.4b A(H5N1) viruses urgently needs to be determined. Furthermore, because virus isolation was unsuccessful, the generation of a recombinant virus carrying these substitutions was necessary to provide matched antigen for serologic testing of the case patient and close contacts.

## Results

### Detection and genetic characterization of the clinical case

The U.S. Centers for Disease Control and Prevention (CDC) confirmed the state of Missouri’s first human case of HPAI A(H5N1) virus infection on September 6, 2024^12^. Although no infectious virus could be recovered from the nasopharyngeal specimen collected from the individual, partial sequences of hemagglutinin (HA) and neuraminidase (NA) genes, along with complete sequences of the matrix (M) and nonstructural (NS) genes of the viral RNA were obtained. A/Missouri/121/2024 was deposited in GenBank (PQ327626–PQ327629) and GISAID (EPI_ISL_19413343) on September 13, 2024^13^. Sequence data revealed two amino acid substitutions in the HA protein: P136S and A156T.

### Generation of recombinant viruses for serologic testing

To facilitate serologic testing and investigate the effects of the HA substitutions identified in A/Missouri/121/2024, we utilized reverse genetics to introduce HA-P136S and HA-A156T mutations, both individually and in combination, into the backbone (PB2, PB1, PA, HA, NP, NA, M and NS gene segments) of A/Texas/37/2024 (TX37), a 2.3.4.4b virus isolated from the first human case associated with the 2024 dairy cattle outbreak^14^. This approach generated four recombinant viruses—rTX37, rTX37-HA-P136S, rTX37-HA-A156T, and rTX37-HA-P136S/A156T—all propagated in Madin-Darby Canine Kidney (MDCK) cells. Serologic testing using rTX37-HA-P136S/A156T demonstrated that the case patient and a household contact had evidence of humoral immune response to A(H5N1) virus. The Microneutralization (MN) assay detected neutralizing antibodies against the recombinant viruses in the index patient and their household contact, while five healthcare workers who were exposed to the patient tested seronegative by MN, hemagglutination inhibition (HI) assay, and the multiplex antibody detection assay^15^.

### Antigenic and neutralization analyses of recombinant viruses

To evaluate the antigenicity of these recombinant viruses, we performed a hemagglutination inhibition (HI) assay with turkey red blood cells (TRBCs) using ferret antisera raised against a panel of recent 2.3.4.4b isolates and a panel of A(H5) candidate vaccine viruses (CVVs) (Table 1). The 2.3.4.4b panel included isolates from 2021 to 2024. The CVV panel included viruses representative of clade 2.3.4.4b, closely related clades 2.3.4.4c and 2.3.4.4 g, and the more distantly related clade 2.3.2.1a^16^. Nearly all antisera effectively inhibited the TX37 isolate, rTX37, and rTX37-HA-P136S, evidenced by ≤2-fold reduced HI titer compared to homologous titers (Table 1). However, introduction of the A156T substitution led to at least a 4-fold reduction in HI titers for all antisera except those raised against TX37, A/Michigan/90/2024 (MI90), and IDCDC-RG80A. In the neutralization assay, antisera to TX37 and MI90 showed significantly reduced neutralizing activity against viruses carrying the A156T substitution, while antisera to IDCDC-RG80A remained effective (Table 2). We observed a similar pattern when we performed the HI assay with horse red blood cells (HRBCs) (Table 3). Some of the differences observed between the TRBC HI and HRBC HI assays may be due to the different sialylglycans expressed on the respective RBC types, leading to different sensitivities to HI. In addition, neutralization assays are generally more sensitive than HI assays and can detect antigenic differences even when antibodies do not inhibit receptor binding. Overall, all three assays demonstrated that the A156T substitution, alone or in combination with P136S, significantly impacted antigenicity.

**Table 1:** Hemagglutination inhibition (HI) assay of recombinant viruses with TRBC^a^

Virus strain name	Clade	Ferret antisera									
		2.3.4.4b	2.3.4.4b	2.3.4.4b	2.3.4.4c	2.3.4.4 g	2.3.2.1a	2.3.4.4b	2.3.4.4b	2.3.4.4b	2.3.4.4b
		IDCDC-RG71A	IDCDC-RG78A	IDCDC-RG80A	IDCDC-RG43A	IDCDC-RG69A	IDCDC-RG63A	AW/2021	Chile/2023	TX37	MI90
**Homologous titer**		320	160	2560	80	80	320	160	40	80	160
**WT TX37 isolate**	**2.3.4.4b**	320	320	2560	320	80	320	160	40	80	160
**rTX37**	**2.3.4.4b**	160	160	2560	320	40	320	160	40	80	160
**rTX37-HA-P136S**	**2.3.4.4b**	160	160	2560	160	40	160	80	20	80	80
**rTX37-HA-A156T**	**2.3.4.4b**	80	20	2560	20	<10	<10	20	10	80	320
**rTX37-HA-P136S/A156T**	**2.3.4.4b**	80	40	2560	10	<10	<10	20	10	80	320

^a^Prototype viruses for CVVs: IDCDC-RG71A (A/Astrakhan/3212/2020), IDCDC-RG78A (A/American Wigeon/South Carolina/22-000345-001/2021), IDCDC-RG80A (A/chicken/Ghana/AVL-763_21VIR7050-39/2021), IDCDC-RG43A (A/gyrfalcon/Washington/41088-6/2014), IDCDC-RG63A (A/duck/Bangladesh/17D1012/2018), IDCDC-RG69A (A/ck/Vietnam/RAHO4-CD-20-421/2020). *AW/2021* A/American Wigeon/South Carolina/22-000345-001/2021, *Chile/2023* A/Chile/25945/2023, *TX37* A/Texas/37/2024, *MI90* A/Michigan/90/2024.

**Table 2:** The neutralization titer 50 (NT50) of ferret antisera against the recombinant viruses by Focus Reduction Assay^a^

Virus strain name	Clade	Ferret antisera									
		2.3.4.4b	2.3.4.4b	2.3.4.4b	2.3.4.4c	2.3.4.4 g	2.3.2.1a	2.3.4.4b	2.3.4.4b	2.3.4.4b	2.3.4.4b
		IDCDC-RG71A	IDCDC-RG78A	IDCDC-RG80A	IDCDC-RG43A	IDCDC-RG69A	IDCDC-RG63A	AW/2021	Chile/2023	TX37	MI90
**Homologous titer**		34995	5888	77384	31421	16767	4972	5037	2275	936	5245
**TX37 isolate**	**2.3.4.4b**	23844 (1.5)	3047 (1.9)	40355 (1.9)	14403 (2.2)	1708 (9.8)	7005 (0.7)	5216 (1.0)	1528 (1.5)	851 (1.1)	5868 (0.9)
**rTX37**	**2.3.4.4b**	23733 (1.5)	3187 (1.8)	36923 (2.1)	12620 (2.5)	2106 (8.0)	8984 (0.6)	5484 (0.9)	2115 (1.1)	868 (1.1)	5554 (0.9)
**rTX37-HA-P136S**	**2.3.4.4b**	22526 (1.6)	2905 (2.0)	35014 (2.2)	14107 (2.2)	1851 (9.1)	6412 (0.8)	4232 (1.2)	1032 (2.2)	689 (1.4)	4223 (1.2)
**rTX37-HA-A156T**	**2.3.4.4b**	5684 (6.2)	357 (16.5)	48574 (1.6)	194 (162.0)	162 (103.5)	<50 (>99.4)	214 (23.5)	<50 (>45.5)	<50 (>18.7)	269 (19.5)
**rTX37-HA-P136S/A156T**	**2.3.4.4b**	4525 (7.7)	390 (15.1)	42615 (1.8)	199 (157.9)	212 (79.1)	<50 (>99.4)	280 (18.0)	62 (36.7)	<50 (>18.7)	380 (13.8)

^a^The value in parentheses represents the fold change between the homologous titer and the neutralization titer of recombinant virus.

**Table 3:** Hemagglutination inhibition (HI) assay of recombinant viruses with HRBC

Virus strain name	Clade	Ferret antisera						
		2.3.4.4b	2.3.4.4b	2.3.4.4b	2.3.4.4c	2.3.4.4b	2.3.4.4b	2.3.4.4b
		IDCDC-RG71A	IDCDC-RG78A	IDCDC-RG80A	IDCDC-RG43A	Chile/2023	TX37	MI90
**Homologous titer**		5120	2560	20480	5120	2560	2560	640
**rTX37**	**2.3.4.4b**	10240	5120	20480	10240	2560	2560	640
**rTX37-HA-P136S/A156T**	**2.3.4.4b**	1280	640	20480	160	160	320	80

### Receptor binding specificity

**Fig. 1:**
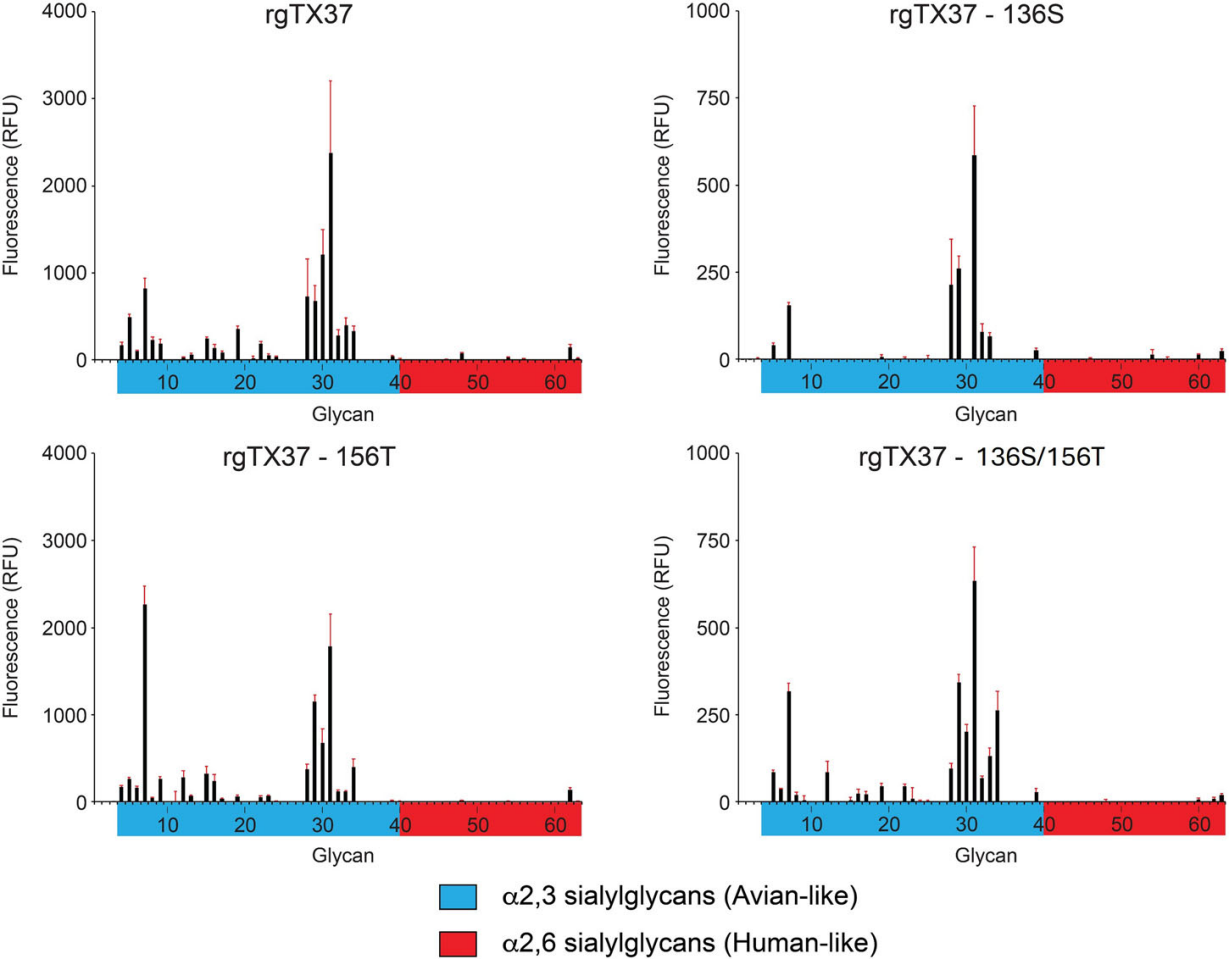
Virus receptor binding assay. Glycan microarray analyses of recombinant viruses. Glycan microarray results for HA of rTX37, rTX37-HA-P136S, rTX37-HA-A156T and rTX37-HA-P136S/A156T. Colored bars distinguish different glycan structures represented on the array. Error bars are standard deviations from six independent replicates on the array. RFU, relative fluorescent units. Each of the numbered glycans’ structures is listed in Supplementary Table 1. Recombinant HA proteins from H5 A/Vietnam/1203/2004 and H3 A/Switzerland/9715293/2013 served as controls for α2,3 and α2,6 receptor binding (Supplementary Fig. 1).

Given the critical role of receptor binding in viral replication and mammalian adaption, we performed a glycan microarray assay to evaluate the sialic acid (SA) receptor binding specificity of the four recombinant viruses (Fig. 1). All four exhibited a strong preference for α2,3-linked (avian-like) SA receptors with minimal binding to α2,6-linked (human-like) SA receptors. Although the substitutions affected the viruses’ binding to a few specific glycans associated with avian-like receptors, they did not enhance the ability of the 2.3.4.4b virus to bind human-like receptors.

### Replicative fitness in primary normal human nasal epithelial cells

**Fig. 2:**
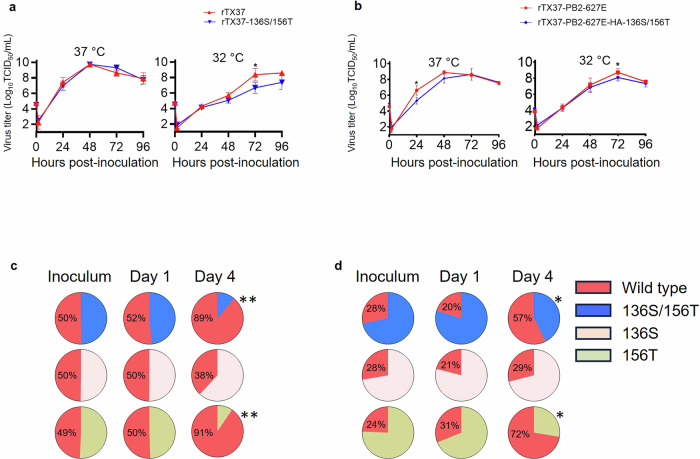
In vitro characterization of viruses carrying the P136S, A156T or P136S/A156T substitutions. **a** Replication kinetics of rTX37 and rTX37-HA-P136S/A156T in NhNE cells at 37 °C and 32 °C. **b** Replication kinetics of rTX37-PB2-627E and rTX37-PB2-627E-HA-P136S/A156T in NhNE cells at 37 °C and 32 °C. **c**, **d** Competition assays in NhNE cells at 32 °C. Virus inocula were prepared by mixing two viruses at 1000 TCID₅₀ with ratios of 1:1 (**c**) and 1:3 (**d**) and used to infect cells in four or six technical replicates. Apical washes were collected daily, and RNA extracted from Day 1 and Day 4 samples was analyzed by next-generation sequencing (NGS). Pie charts show the mean frequencies of HA variants in apical wash samples: wild type (red), P136S/A156T (blue), P136S (pink) and A156T (sage). The average percentage of wild-type virus is shown in the figure. Statistical analysis of wild-type virus frequencies between the inoculum and samples collected on days 1 and day 4 was performed using one-way ANOVA followed by Dunnett’s multiple comparisons test. Error bars indicate SD. “*” indicates significant difference (*P* < 0.05). “**” indicates very significant difference (*P* < 0.01).

To assess the replication capacity of viruses with low binding affinity for human-like receptors in the human upper respiratory tract, we analyzed viral replication kinetics in primary normal human nasal epithelial cells (NhNE), which mimic the pseudostratified epithelium of the upper respiratory tract. At 37 °C, both rTX37 and rTX37-HA-P136S/A156T replicated robustly and reached peak titer of 10^9.5^ TCID_50_/mL by 48 h post inoculation (hpi). To simulate the temperature of the human nasal cavity, we repeated the experiment at 32 °C and observed slower replication for both viruses; by 72 hpi rTX37 reached only 10^8.3^ TCID_50_/mL, whereas rTX37-HA-P136S/A156T replicated to only 10^6.7^ TCID_50_/mL, a significantly lower titer compared to rTX37 (*P* = 0.02) (Fig. 2a).

PB2 residue 627 is a well-known determinant of mammalian adaption^17,18^. TX37 carries a lysine at this residue (PB2-627K), while some other clade 2.3.4.4b viruses from humans carry a glutamic acid (PB2-627E). Because the PB2 sequence of A/Missouri/121/2024 could not be obtained from the low levels of viral RNA present in the clinical specimen, we considered it beneficial to assess the impact of HA-P136S/A156T in a PB2-627E background. We introduced a PB2-K627E substitution into rTX37, rTX37-HA-P136S, rTX37-HA-A156T and rTX37-HA-P136S/A156T, generating rTX37-PB2-627E, rTX37-PB2-627E-HA-P136S, rTX37-PB2-627E-HA-A156T and rTX37-PB2-627E-HA-P136S/A156T, respectively. At 37 °C, rTX37-PB2-627E-HA-P136S/A156T replicated less efficiently than rTX37-PB2-627E at 24 hpi (*P* = 0.02), and at 32 °C, the mutant also showed reduced replication at 72 hpi (*P* = 0.04) (Fig. 2b).

The slightly reduced replication efficiency of the mutant virus may lead to a competitive disadvantage if it emerges in an immunologically naïve host. To assess replicative fitness directly, we conducted a competition assay in NhNE cells at 32 °C by co-infecting cells with virus mixtures at defined ratios. The ratios were determined by next-generation sequencing (NGS) analysis of nucleotide frequencies corresponding to HA mutations P136S and/or A156T, as shown in Fig. 2. In co-infections at a 1:1 ratio [wild-type (rTX37-PB2-627E): mutant], the wild-type virus outcompeted both the P136S/A156T and A156T mutants and reached 89% and 91% of the population by day 4, respectively (Fig. 2c). When the initial ratio was increased to 1:3 (wild-type: mutant), the wild-type virus still predominated, reaching 57% and 72% of the viral population by day 4 in co-infections with the P136S/A156T and A156T mutants, respectively (Fig. 2d). These findings suggest that the A156T substitution, alone or in combination with P136S, reduces viral fitness in human nasal epithelial cells.

### Replicative fitness in ferrets

**Fig. 3:**
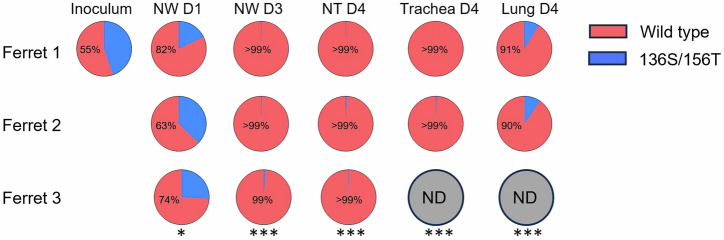
Competition of wild-type virus and P136S/A156T mutant virus in a ferret model. Each ferret was inoculated with 10⁴ TCID₅₀ of mixed rTX37-PB2-627E (wild type) and rTX37-PB2-627E-HA-P136S/A156T. Nasal wash (NW) samples were collected from three ferrets on days 1 (D1) and day 3 (D3) post-inoculation. Nasal turbinate (NT), Trachea and lung samples were collected from the ferrets on day 4 (D4) post-inoculation. RNA extracted from samples was analyzed by next-generation sequencing (NGS). Pie charts show the mean frequencies of HA variants in samples: wild type (red) and P136S/A156T (blue). The percentage of wild-type virus is indicated in the figure. “ND” indicates not detected. Statistical analysis of wild-type virus frequencies between the inoculum and different ferret sample groups was performed using one-way ANOVA followed by Dunnett’s multiple comparisons test. “*” indicates a significant difference (*P* < 0.05); “***” indicates a highly significant difference (*P* < 0.001).

We further conducted a competition study in ferrets using a 1:1 mixture of rTX37-PB2-627E (wild-type) and rTX37-PB2-627E-HA-P136S/A156T (mutant). The ratio of the mixture was determined by NGS analysis of nucleotide frequencies corresponding to HA mutations P136S and A156T, as shown in Fig. 3. Each ferret was intranasally inoculated with 10⁴ TCID₅₀ of the virus mixture. Nasal wash (NW) samples were collected on days 1 and 3 post-inoculation, and tissue samples were collected from three ferrets on day 4. Compared to the inoculum, the proportion of the wild-type virus in NW increased in all three ferrets by day 1 and reached at or over 99% by day 3 (Fig. 3). On day 4, the wild-type virus predominated in nasal turbinates (>99%), trachea (>99%), and lungs (≥90%). These findings align with the reduced replication efficiency of the HA-P136S/A156T mutant observed in vitro and suggest that these HA substitutions impair viral fitness in the respiratory tract.

## Discussion

In this study, reverse genetics proved an invaluable tool to characterize naturally occurring mutations in influenza viruses that could not be recovered from clinical specimens. From the time the partial HA sequence was obtained on September 13, 2024, to the completion of plasmid construction, virus rescue, and the first HI results on September 23, only 10 calendar days (6 business days) had elapsed. The reverse genetics-generated virus also proved important for serologic testing of the Missouri patient and close contacts using an optimally matched antigen to ensure HI and neutralization results were accurate. Reverse genetics can be an important tool for public health, and its rapid turnaround time can be especially valuable when speed is essential, such as in responding to influenza outbreaks and assessing the pandemic potential of emerging influenza viruses.

The HA substitution A156T has previously been reported in clade 2.3.4.4b A(H5N1) viruses from wild birds in Canada and Norway, but it was not found in over 2000 Canadian clade 2.3.4.4b A(H5N1) isolates^19^ and was rarely detected in A(H5N1) viruses associated with the U.S. cattle outbreaks. The P136S/A156T substitutions identified in the Missouri human case may have emerged independently under distinct evolutionary pressures in the human host. Therefore, further characterization of the antigenic and fitness impact of these two substitutions in the clade 2.3.4.4b A(H5N1) background is warranted.

The HA P136S/A156T substitutions altered the antigenicity of the 2.3.4.4b A(H5N1) virus, most likely through the introduction of an N-linked glycosylation site at residue 154 enabled by the A156T substitution. This glycosylation likely shields antigenic site B from antibody recognition, resulting in reduced HI and neutralization titers, consistent with previous studies showing that A156T contributes to antigenic change^4,19,20^. The A156T substitution did not alter receptor-binding specificity from avian-like α2,3-SA to human-like α2,6-SA. However, both P136S and A156T substitutions affected binding to a few specific glycans associated with avian-like receptors. Whether these changes are associated with the reduced fitness of the rTX37-HA-P136S/A156T mutant virus remains to be determined.

The in vitro and in vivo data reveal a reduction in viral replicative fitness in human nasal epithelial cells and in ferrets, with or without P136S, indicating a potential trade-off between immune escape and viral fitness. Additionally, the HA changes may have reduced viral fitness by affecting other HA-associated properties, such as stability, fusion pH, or the balance between HA and NA functions^5,19–22^. A limitation of this study is that the two HA substitutions (P136S and A156T) were characterized in the TX37 background rather than in the A/Missouri/121/2024 (MO121) background. This was necessary because the wild-type virus could not be isolated and only partial HA and NA sequences, along with the full M and NS segments, were obtained from the clinical specimen. Previous studies have shown that the phenotypic effects of specific mutations in influenza viruses can vary depending on the genetic background; therefore, it is possible that these substitutions may behave differently in the full MO121 background. Nevertheless, based on the partial sequences available, the MO121 virus appears closely related to other viruses associated with the current cattle outbreak. Comparison of the available MO121 HA sequence to other human 2.3.4.4b viruses associated with the outbreak identified only these two substitutions relative to TX37, which appears to be one of the closest HA sequences (Supplementary Table 5). The NA sequence of MO121 is also incomplete, with amino acids 285–404 missing; however, in the regions that are available, MO121 NA differs from TX37 by only two amino acids (Supplementary Table 6). For these reasons, introducing the P136S and A156T substitutions into the TX37 background represents the best available strategy to characterize their functional impact and assess their potential public health risk should they emerge in clade 2.3.4.4b A(H5N1) viruses.

Importantly, the IDCDC-RG80A candidate vaccine virus (CVV), which already contains a threonine at residue 156, demonstrated promise as an effective vaccine candidate against clade 2.3.4.4b viruses both with and without the A156T substitution. These findings underscore the importance of maintaining a diverse and up-to-date library of A(H5) CVVs with broad antigenic coverage to support pandemic preparedness. Continued surveillance and rapid characterization of emerging A(H5) viruses like A/Missouri/121/2024 are essential to timely update CVVs prior to sustained human transmission.

## Methods

### Biosafety and animal ethics

All experiments involving highly pathogenic avian influenza (HPAI) A(H5N1) viruses were conducted in Biosafety Level 3 enhanced (BSL-3E) or Animal Biosafety Level 3 (ABSL-3) laboratories at the U.S. Centers for Disease Control and Prevention (CDC), including enhancements required by the U.S. Department of Agriculture and the Federal Select Agent Program. Personnel performing virus-related work received comprehensive training in biosafety protocols and procedural techniques, with competency assessed prior to laboratory access. Recombinant DNA work was approved by the CDC Institutional Biosafety Committee (IBC).

All ferret experiments were approved by the CDC’s Institutional Animal Care and Use Committee (IACUC) and conducted in an Association for Assessment and Accreditation of Laboratory Animal Care (AAALAC) International-accredited ABSL-3 facility. Procedures complied with CDC policies and U.S. federal regulations on the ethical treatment of animals in research. All efforts were made to minimize animal distress, and the number of animals used was limited to the minimum necessary for obtaining scientifically valid results.

### Cells and viruses

Madin-Darby Canine Kidney (MDCK) cells, human embryonic kidney 293 T (HEK293T) cells, African green monkey kidney cells (Vero cells) and 9–10-day-old embryonated chicken eggs were used for virus propagation and rescue. MDCK cells were maintained in Minimum Essential Medium (MEM) supplemented with 5% fetal bovine serum (FBS), and HEK293T cells were cultured in Dulbecco’s Modified Eagle Medium–High Glucose (DMEM-High Glucose) supplemented with 10% FBS. Embryonated chicken eggs were incubated at 35 °C and candled prior to inoculation to ensure the embryo viability. Wild type influenza A/Texas/37/2024, A/Chile/25945/2023 and A/Michigan/90/2024 viruses were propagated in MDCK cells. Wild type influenza A/American Wigeon/South Carolina/22-000345-001/2021 viruses were propagated in embryonated chicken eggs. Candidate vaccine viruses (CVVs) were rescued in Vero cells with the donor of PB2, PB1, HA, NP, NA, M and NS gene segments from A/Puerto Rico/8/1934 (PR8) influenza virus. Recombinant viruses (rTX37, rTX37-HA-P136S, rTX37-HA-A156T, rTX37-HA-P136S/A156T, rTX37-PB2-627E, rTX37-PB2-627E-HA-P136S, rTX37-PB2-627E-HA-A156T, and rTX37-PB2-627E-HA-P136S/A156T) were rescued in HEK239T and MDCK coculture with the backbone (PB2, PB1, PA, HA, NP, NA, M and NS gene segments) of TX37, and propagated in MDCK cells as described in refs. ^23–26^.

### Immunization of ferrets and preparation of antisera

Ferret antisera against A(H5) viruses were produced previously as part of the CDC Influenza Division’s surveillance program and were not generated specifically for this study. Generally, outbred ferrets (Triple F Farms) seronegative for currently circulating strains of influenza A and B were intranasally inoculated with live CVV or wild type influenza viruses at different dosage, or subcutaneous injection with live or inactivated virus. Antisera were collected 14 days post infection to determine hemagglutination inhibition (HI) titer. If HI titer was ≤40, each ferret received a subcutaneous injection in both hind legs with a whole virion antigen preparation containing at least 1024 hemagglutination units (HAU) of virus mixed with Titermax Gold Adjuvant (Sigma-Aldrich, MO, USA). Antisera were then collected 14 days post-injection. For the sera used in this study, antisera against IDCDC-RG78A, A/Chile/25945/2023 and A/Michigan/90/2024 were generated without boost, while sera against IDCDC-RG71A, IDCDC-RG80A, IDCDC-RG43A, IDCDC-RG69A, IDCDC-RG63A, A/American Wigeon/South Carolina/22-000345-001/2021 and TX37 were generated with boost. TX37 antisera generation utilized subcutaneously injected inactivated virus for both priming and boosting due to its high pathogenicity in ferrets^27^.

### Hemagglutination inhibition assay

Ferret antisera were treated with receptor-destroying enzyme (RDE) overnight at 37 °C, then heat-inactivated at 56 °C for 30 min. The RDE-treated antisera were adsorbed with packed turkey red blood cells (TRBCs) or horse red blood cells (HRBCs) to remove nonspecific agglutinins. Treated ferret antisera were tested in HI assay with TRBCs and HRBCs. Briefly, 4 HAU of virus in 25 μL was mixed with 25 μL of two-fold serially diluted antiserum. After a 30 min incubation at room temperature, 50 μL of 0.5% TRBCs or 1% HRBCs were added and allowed to settle for 30 min (TRBCs) or 60 min (HRBCs). Wells were observed for hemagglutination activity and the reciprocal of the highest antiserum dilution that completely inhibited hemagglutination was recorded as the HI titer.

### Focus reduction assay

The focus reduction assay was performed as previously described in ref. ^28^. Briefly, 2-fold serially diluted antiserum samples and 600 focus-forming units of virus were added to 96-well plates containing confluent MDCK cells and incubated at 37 °C for 2 h. An equal volume of overlay, consisting of 1.2% Avicel RC/CL in 2X MEM with 1 μg/mL TPCK-treated trypsin, 0.1% bovine serum albumin (BSA) fraction V, and antibiotics, was then added. After 14 h of incubation, the cells were fixed with 70% cold ethanol at 4 °C for 30 min. After fixation, the fluorogenic NA substrate (BTP3-Neu5Ac Na)^29^ was added to washed reaction plates to visualize the number of foci. The values of 50% neutralization (NT50) were calculated in GraphPad Prism 10.

### Virus receptor binding assay

Glycan microarray analyses have been described previously^30^. β-Propiolactone (BPL)-inactivated concentrated viruses were used in this assay instead of recombinant proteins. Briefly, the inactivated viruses were mixed and incubated with ferret antisera raised against IDCDC-RG80A, followed by labeling with mouse anti-ferret antibody. After incubating on ice for 60 min, the mixtures were applied to the microarray slides. The slides were washed and dried before being scanned for fluorescence and spot intensities were quantified and analyzed. Supplementary Table 1 lists the specific glycans present on the array.

### Infection of NhNE cells

Primary human nasal epithelial cell cultures were obtained from Epithelix and cultured under air–liquid interface conditions that resemble the pseudostratified epithelial lining of the human respiratory epithelium. Cells were washed twice before infection with TEER buffer, then infected with viruses at 1000 TCID_50_/well or were mock-infected with medium only. Infected cells were maintained respectively at 37 °C and 32 °C. At 2 h, 24 h, 48 h, 72 h and 96 h post-infection, supernatants were collected and titrated by TCID_50_ assay. For competition experiments, NhNE cells were inoculated with 1:1 or 1:3 mixtures of rTX37-PB2-627E and rTX37-PB2-627E-HA-P136S, rTX37-PB2-627E and rTX37-PB2-627E-HA-A156T, or rTX37-PB2-627E and rTX37-PB2-627E-HA-P136S/A156T, at 1000 TCID₅₀ per well. Four replicated wells per condition. The ratios were determined by next-generation sequencing (NGS) analysis of nucleotide frequencies corresponding to HA mutations P136S and/or A156T. After incubation at 37 °C for 1 h, the inoculum was removed, cells were washed, and subsequently incubated at 32 °C. Apical washes were collected daily for 4 days.

### Infection of ferrets

Three 17-month-old male ferrets (Triple F Farms) that were seronegative for seasonal influenza A and B viruses, were housed in temperature- and humidity-controlled ABSL-3 rooms with a 12 h light/dark cycle. Group size of three (only one group in this study) was selected to use the minimum number of animals required to achieve scientific validity. Blinding was not applied to the animal study part as there was only one group of three animals.

Each ferret was anesthetized by intramuscular injection of a ketamine/xylazine mixture followed by intranasal inoculation with a 1:1 mixture of rTX37-PB2-627E (wild-type) and rTX37-PB2-627E-HA-P136S/A156T mutant virus at a total dose of 10⁴ TCID_50_ per ferret. The input virus ratio was determined by Illumina next-generation sequencing (NGS) analysis of nucleotide frequencies corresponding to HA mutations P136S and/or A156T. Nasal wash samples were collected from three ferrets on days 1 and 3 post-infection. On day 4, the three ferrets were euthanized for collection of nasal turbinate, trachea, and lung tissues. All animals enrolled in the study completed the experiments as planned. No animals or data points were excluded from the analysis.

Animals were monitored twice daily using a standardized humane endpoint scoring system. Scoring criteria included: 2 points each for hunched posture/huddling, piloerection/ruffled coat, hair loss; 3 points each for dehydration or abnormal temperature; 5 points each for respiratory distress or anemia; 10 points each for paralysis, torticollis, moribund state, unresponsiveness, ≥20% weight loss, hypothermia (>3 days), hemorrhage, traumatic injury requiring intervention, or other veterinary concerns. Ferrets reaching a total score ≥10 were humanely euthanized according to AVMA guidelines. None of the ferrets reached humane point in this study.

### Sequencing of A/Missouri/121/2024 clinical specimen

Viral RNA was extracted from nasopharyngeal specimen using the QIAamp Viral RNA Mini Kit (Qiagen). RNA was amplified with the universal influenza A primer set^23^, and with segment-specific primers for HA and NA. cDNA was also generated from undiluted RNA using the Twist Total Nucleic Acids Library Preparation EF Kit 2.0 (Twist Bioscience).

Indexed libraries were prepared from amplicons and cDNA, pooled by size and concentration, and enriched with the Twist Comprehensive Viral Panel probe set using the Fast Hybridization protocol. Sequencing was performed on an Illumina MiSeq (300-cycle v2 kit).

Reads from all enriched libraries were combined for analysis. The minimum average segment coverage was >9,000×. Bases with <25× coverage were masked as “N” upon public release; all other bases met the ≥25× coverage threshold. Additionally, the coverage depth of each segment is as blow: HA, 9293; NA, 60139; MP, 200226; NS, 48029.

### Sequencing of tissue culture and animal samples

Viral RNA was extracted from apical washes, ferret organ and nasal wash samples or inoculum using QIAamp Viral RNA Mini Kit (QIAGEN) according to the manufacturers’ protocols. The HA gene was amplified using PCR reaction. In brief, the SuperScript™ IV One-Step RT-PCR System (ThermoFisher) was used to amplify the full length of the HA gene. The RT–PCR was performed in a 50 μL reaction using 5–10 μL of RNA. The cycling parameters were as follows: 50 °C for 45 min, 55 °C for 10 min, 94 °C for 2 min; 5 cycles of 94 °C for 30 s, 65 °C for 30 s, and 68 °C for 3 min; followed by 33 cycles of 94 °C for 30 s, 63 °C for 30 s, and 68 °C for 2 min; and a final elongation step at 68 °C for 10 min.

DNA sequencing libraries were prepared using the Nextera DNA Flex Library Kit (Illumina) and sequenced on the MiniSeq System (Illumina). Inoculum samples from NhNE and ferret infection experiments were sequenced in duplicate. All apical wash samples collected at different time points were sequenced once, as four biological replicates were included for each condition (Supplementary Tables 2 and 3). Ferret nasal wash and tissue samples were sequenced once, except in cases of low coverage, in which samples were resequenced (Supplementary Table 4). NGS data were analyzed using CLC Genomics Workbench (Qiagen). Variant frequencies for P136S and A156T were calculated from mapped reads. Variant detection parameters were set at a minimum coverage of 1,000 reads and a detection threshold of 1%. Values below 1% in the supplementary tables are provided for reference only and have not been validated. In cases of low coverage, variant frequencies were read manually from the read tracks; these values have not been validated and are provided for reference purposes.

### Statistical analysis

Replication kinetics were analyzed using two-way analysis of variance (ANOVA). Comparisons between virus groups at individual time points were performed using Tukey’s multiple comparisons test. For each virus and time point, 4–6 technical replicates were analyzed. Competition experiments in NhNE were analyzed using one-way ANOVA, with Dunnett’s multiple comparisons test used to compare wild-type virus frequencies between the inoculum and samples collected from different days. Similarly, competition experiments in ferret models were analyzed using one-way ANOVA, with Dunnett’s multiple comparisons test used to compare wild-type virus frequencies between the inoculum and different ferret tissue or nasal wash samples. Statistical significance was defined as *P* < 0.05. All analyses were conducted using GraphPad Prism version 10.

## Supplementary information

Supplementary Figure and Tables

Arrive essential 10 checklist

## Data Availability

The datasets used in the current study are available within the manuscript and the Supplementary file. Any additional data supporting the findings of this study are available from the corresponding author upon request.

## Code availability

No custom code or scripts were used to generate or analyze datasets. Full details of the statistical methods used for figure generation are provided in “Statistical Analysis”. Analyses were conducted using ClustalW multiple alignment in BioEdit software and GraphPad Prism version 10.
