# Supplementary material for: Impact of naturally occurring hemagglutinin substitutions on antigenicity and fitness of influenza A(H5N1) virus
Source: Npj Viruses. 2025 Oct 2;3:72. doi: 10.1038/s44298-025-00154-5 (PMC12489106; doi:10.1038/s44298-025-00154-5)
Supplement: Supplementary file 1 — Supplementary Figure and Tables [file 44298_2025_154_MOESM1_ESM.pdf]

Supplementary Figure and Tables

Supplementary Figure 1: Glycan microarray controls

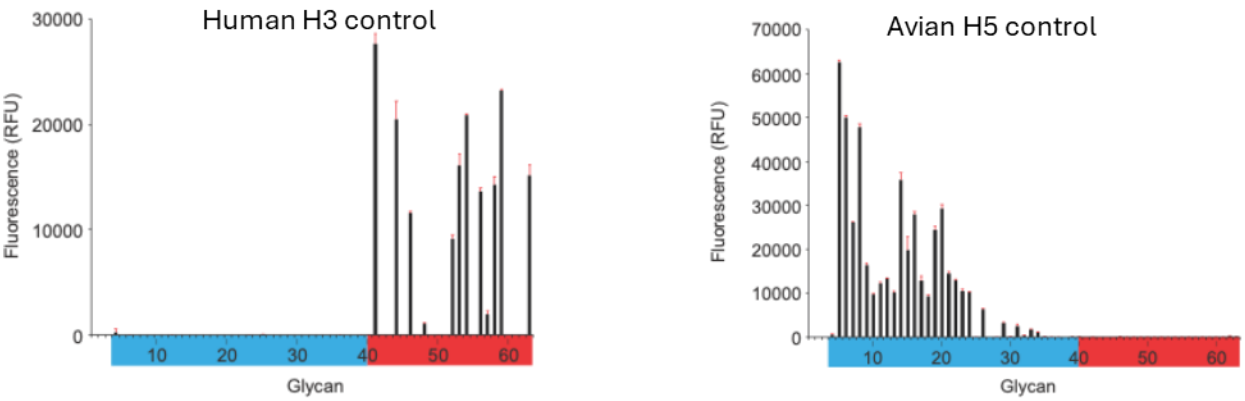

Glycan microarray analyses of recombinant HA protein from H5 A/Vietnam/1203/2004 and H3 A/Switzerland/9715293/2013. They served as controls for α2,3 and α2,6 receptor binding. Colored bars distinguish different glycan structures represented on the array. Error bars are standard deviations from six independent replicates on the array. RFU, relative fluorescent units. Each of the numbered glycans' structures is listed in Supplementary Table 1.

.

Supplementary Table 1: Numbered glycans' structures

Glycan type	Structure	Description
Sialic acid		
1	α-Neu5Ac–Sp8	α-Neu5Ac
2	α-Neu5Ac–Sp11	α-Neu5Ac
3	β-Neu5Ac-Sp8	β-Neu5Ac
**α2-3 sialosides**		
4	Neu5Acα2-3(6-O-Su)Galβ1-4(Fucα1-3)GlcNAcβ–Sp8	α2-3 so4
5	Neu5Acα2-3Galβ1-3(6OSO3)GalNAcα-Sp8	α2-3 so4
6	Neu5Acα2-3Galβ1-4(6OSO3)GlcNAcβ-Sp8	α2-3 so4
7	Neu5Acα2-3Galβ1-4(Fucα1-3)(6OSO3)GlcNAcβ–Sp8	α2-3 so4
8	Neu5Acα2-3Galβ1-3(6OSO3)GlcNAc-Sp8	α2-3 so4
9	Neu5Acα2-3Galβ1-3(Neu5Acα2-3Galβ1-4)GlcNAcβ-Sp8	di-sialoside
10	Neu5Acα2-3Galβ1-3(Neu5Acα2-3Galβ1-4GlcNAcβ1-6)GalNAc–Sp14	di-sialoside
11	Neu5Acα2-3Galβ1-4GlcNAcβ1-2Manα1-3(Neu5Acα2-3Galβ1-4GlcNAcβ12Manα1-6)Manβ1-4GlcNAcβ1-4GlcNAcβ-Sp12	α2-3 biantennary
12	Neu5Acα2-3Galβ-Sp8	α2-3
13	Neu5Acα2-3GalNAcα–Sp8	α2-3
14	Neu5Acα2-3Galβ1-3GalNAcα-Sp8	α2-3
15	Neu5Acα2-3Galβ1-3GlcNAcβ–Sp0	α2-3
16	Neu5Acα2-3Galβ1-3GlcNAcβ–Sp8	α2-3
17	Neu5Acα2-3Galβ1-4Glcβ–Sp0	α2-3
18	Neu5Acα2-3Galβ1-4Glcβ–Sp8	α2-3
19	Neu5Acα2-3Galβ1-4GlcNAcβ–Sp0	α2-3
20	Neu5Acα2-3Galβ1-4GlcNAcβ–Sp8	α2-3
21	Neu5Acα2-3GalNAcβ1-4GlcNAcβ-Sp0	α2-3
22	Neu5Acα2-3Galβ1-4GlcNAcβ1-3Galβ1-4GlcNAcβ-Sp0	α2-3
23	Neu5Acα2-3Galβ1-3GlcNAcβ1-3Galβ1-3GlcNAcβ-Sp0	α2-3
24	Neu5Acα2-3Galβ1-4GlcNAcβ1-3Galβ1-4GlcNAcβ1-3Galβ1-4GlcNAcβ– Sp0	α2-3
25	Neu5Acα2-3Galβ1-4GlcNAcβ1-3Galβ1-3GlcNAcβ-Sp0	α2-3
26	Neu5Acα2-3Galβ1-3GalNAc–Sp14	α2-3
27	Neu5Acα2-3Galβ1-4(Fucα1-3)GlcNAcβ1-6(Galβ1-3)GalNAc–Sp14	α2-3 fucosylated
28	Neu5Acα2-3Galβ1-3(Fucα1-4)GlcNAcβ–Sp8	α2-3 fucosylated
29	Neu5Acα2-3Galβ1-4(Fucα1-3)GlcNAcβ–Sp0	α2-3 fucosylated

30	Neu5Acα2-3Galβ1-4(Fucα1-3)GlcNAcβ–Sp8	α2-3 fucosylated
31	Neu5Acα2-3Galβ1-4(Fucα1-3)GlcNAcβ1-3Galβ-Sp8	α2-3 fucosylated
32	Neu5Acα2-3-Galβ1-3(Fucα1-4)GlcNAcβ1-3Galβ1-4(Fucα1-3)GlcNAcβ-Sp0	α2-3 fucosylated
33	Neu5Acα2-3Galβ1-4(Fucα1-3)GlcNAcβ1-3Galβ1-4(Fuca1-3)GlcNAcβ-Sp0	α2-3 fucosylated
34	Neu5Aca2-3Galβ1-4(Fuca1-3)GlcNAcβ1-3Galβ1-4(Fuca1-3)GlcNAcβ1-3Galβ1-4(Fuca1-3)GlcNAcβ-Sp0	α2-3 fucosylated
35	Neu5Acα2-3(GalNAcβ1-4)Galβ1-4GlcNAcβ-Sp0	α2-3 internal
36	Neu5Acα2-3(GalNAcβ1-4)Galβ1-4GlcNAcβ-Sp8	α2-3 internal
37	Neu5Acα2-3(GalNAcβ1-4)Galβ1-4Glcβ–Sp0	α2-3 internal
38	Neu5Acα2-3(Galβ1-3GalNAcβ1-4)Galβ1-4Glcβ-Sp0	α2-3 internal
39	Neu5Acα2-3(Fucα1-2Galβ1-3GalNAcβ1-4)Galβ1-4Glcβ-Sp0	α2-3 internal
40	Neu5Acα2-3(Fucα1-2Galβ1-3GalNAcβ1-4)Galβ1-4Glcβ-Sp9	α2-3 internal
**α2-6 sialosides**		
41	Neu5Acα2-6Galβ1-4[6OSO3]GlcNAcβ-Sp8	α2-6 so4
42	Neu5Acα2-6Galβ1-4GlcNAcβ1-2Manα16(Galβ1-4GlcNAcβ1-2Manα1-3)Manβ1-4GlcNAcβ1-4GlcNAcβ-Sp12	α2-6 branched
43	Neu5Acα2-6Galβ1-4GlcNAcβ1-2Manα1-3(Neu5Acα2-6Galβ1-4GlcNAcβ1-2Manα1-6)Manβ1-4GlcNAcβ1-4GlcNAcβ-Sp12	α2-6 biantenary
44	NeuAcα(2-6)-Galβ(1-4)-GlcNAcβ(1-3)-Galβ(1-4)-GlcNAcβ(1-2)-Manα(1-3)-[NeuAcα(2-6)-Galβ(1-4)-GlcNAcβ(1-3)-Galβ(1-4)-GlcNAcβ(1-2)-Manα(1-6)]-Manβ(1-4)-GlcNAcβ(1-4)-GlcNAcβ-Sp12	α2-6 biantenary
45	Neu5Acα2-6Galβ1-4GlcNAcβ1-3Galβ1-4GlcNAcβ1-3Galβ1-4GlcNAcβ1-2Manα1-3(Neu5Acα2-6Galβ1-4GlcNAcβ1-3Galβ1-4GlcNAcβ1-3Galβ1-4GlcNAcβ1-2Manα1-6)Manβ1-4GlcNAcβ1-4GlcNAcβ-Sp12	α2-6 biantenary
46	Neu5Acα2-6Galβ1-4GlcNAcβ1-3Galβ1-4GlcNAcβ(1-3)(Neu5Acα2-6Galβ1-4GlcNAcβ1-3Galβ1-4GlcNAcβ1-6)GalNAca-Sp14	α2-6 biantenary
47	Neu5Acα2-6Galβ1-4GlcNAcβ1-2Manα1-3(Neu5Acα2-6Galβ1-4GlcNAcβ12Manα1-6)Manβ1-4GlcNAcβ1-4GlcNAcβ-Sp8	α2-6 biantenary
48	Neu5Acα2-6Galβ1-4GlcNAcβ1-2Manα1-3(Neu5Acα2-6Galβ1-4GlcNAcβ12Manα1-6)Manβ1-4GlcNAcβ1-4GlcNAcβ-Sp12	α2-6 biantenary
49	Neu5Acα2-6Galβ1-4GlcNAcβ1-2Manα1-3(Galβ1-4GlcNAcβ1-2Manα16)Manβ1-4GlcNAcβ1-4GlcNAcβ-Sp12	α2-6 biantenary
50	Neu5Acα2-6GalNAcα–Sp8	α2-6
51	Neu5Acα2-6Galβ–Sp8	α2-6
52	Neu5Acα2-6Galβ1-4Glcβ–Sp8	α2-6
53	Neu5Acα2-6Galβ1-4GlcNAcβ–Sp0	α2-6
54	Neu5Acα2-6Galβ1-4GlcNAcβ–Sp8	α2-6
55	Neu5Acα2-6GalNAcβ1-4GlcNAcβ-Sp0	α2-6
56	Neu5Acα2-6Galβ1-4GlcNAcβ1-3Galβ1-4GlcNAcβ-Sp0	α2-6
57	Neu5Acα2-6Galβ1-4GlcNAcβ1-3Galβ1-4GlcNAcβ1-3GalNAca-Sp14	α2-6

58	Neu5Aca2-6Galβ1-4GlcNAcβ1-3Galβ1-4GlcNAcβ1-3Galβ1-4GlcNAcβ-Sp0	α2-6
59	Neu5Acα2-6Galβ1-4GlcNAcβ1-3Galβ1-4(Fucα1-3)GlcNAcβ1-3Galβ1-4(Fucα1-3)GlcNAcβ-Sp0	α2-6 fucosylated
60	Neu5Acα2-6(Galβ1-3)GlcNAcβ1-4Galβ1-4Glcβ-Sp10	α2-6 internal
61	Neu5Acα2-6(Galβ1-3)GalNAca-Sp14	α2-6 internal
62	Neu5Acα2-6Galβ1-4GlcNAcβ1-6(Galβ1-3)GalNAca-Sp14	α2-6 internal
63	NeuAcα2-6Galβ1-4GlcNAcβ1-3Galβ1-4GlcNAcβ1-6(Galβ(1-3)GalNAca-Sp14	α2-6 internal

Supplementary Table 2: NGS summary of NhNE samples with 1:1 infection ratio

Sample (WT:Mutant=1:1)	136S			156T		
	Variants Count	Coverage	Frequency	Variants Count	Coverage	Frequency
Inoculum-WT:P136S-repeat 1	6836	13523	50.55%			
Inoculum-WT:P136S-repeat 2	2758	5535	49.83%			
Inoculum-WT:A156T-repeat 1				8872	16720	53.06%
Inoculum-WT:A156T-repeat 2				3233	6701	48.25%
Inoculum-WT:136S/156T-repeat 1	5098	10358	49.22%	4815	9546	50.44%
Inoculum-WT:136S/156T-repeat 2	3840	7817	49.12%	4005	7920	50.57%
D1-WT:P136S_well 1	3503	10102	34.68%			
D1-WT:P136S_well 2	6467	15149	42.69%			
D1-WT:P136S_well 3	7531	15276	49.30%			
D1-WT:P136S_well 4	2935	3962	74.08%			
D1-WT:A156T_well 1				2234	3312	67.45%
D1-WT:A156T_well 2				2251	7733	29.11%
D1-WT:A156T_well 3				4227	8114	52.10%
D1-WT:A156T_well 4				3781	7692	49.15%
D1-WT:136S/156T_well 1	2218	4949	44.82%	2278	5009	45.48%
D1-WT:136S/156T_well 2	1017	2485	40.93%	1049	2516	41.69%
D1-WT:136S/156T_well 3	532	1589	33.48%	524	1571	33.35%
D1-WT:136S/156T_well 4	461	634*	72.71%	479	637*	75.20%
D4-WT:P136S_well 1	2513	6425	39.11%			
D4-WT:P136S_well 2	4022	8770	45.86%			
D4-WT:P136S_well 3	5200	6142	84.66%			
D4-WT:P136S_well 4	6226	7962	78.20%			
D4-WT:A156T_well 1				271	2078	13.04%
D4-WT:A156T_well 2				177	2450	7.22%
D4-WT:A156T_well 3				641	6820	9.40%
D4-WT:A156T_well 4				293	3560	8.23%
D4-WT:136S/156T_well 1	44	3185	1.38%	45	3138	1.43%
D4-WT:136S/156T_well 2	137	972*	14.09%	126	901*	13.98%
D4-WT:136S/156T_well 3	197	1562	12.61%	187	1560	11.99%
D4-WT:136S/156T_well 4	219	1329	16.48%	224	1379	16.24%

*: The data with lower coverage and threshold were read manually from reads tracks.

Supplementary Table 3: NGS summary of NhNE samples with 1:3 infection ratio

	136S			156T		
Sample (WT:Mutant=1:3)	Variants Count	Coverage	Frequency	Variants Count	Coverage	Frequency
Inoculum-WT:P136S-repeat 1	6323	8820	71.69%			
Inoculum-WT:P136S-repeat 2	2192	3008	72.87%			
Inoculum-WT:A156T-repeat 1				7583	9822	77.20%
Inoculum-WT:A156T-repeat 2				2838	3828	74.14%
Inoculum-WT:136S/156T-repeat 1	8569	13359	64.14%	8979	13710	65.49%
Inoculum-WT:136S/156T-repeat 2	3894	4932	78.95%	3997	5116	78.13%
D1-WT:P136S_well 1	2521	3237	77.88%			
D1-WT:P136S_well 2	3268	4045	80.79%			
D1-WT:P136S_well 3	7907	11247	70.30%			
D1-WT:P136S_well 4	9601	11046	86.92%			
D1-WT:A156T_well 1				9092	11012	82.56%
D1-WT:A156T_well 2				4149	6176	67.18%
D1-WT:A156T_well 3				862	1535	56.16%
D1-WT:A156T_well 4				342	491	69.65%
D1-WT:136S/156T_well 1	2733	3319	82.34%	2816	3446	81.72%
D1-WT:136S/156T_well 2	4718	5590	84.40%	5237	6181	84.73%
D1-WT:136S/156T_well 3	4480	6379	70.23%	4533	6407	70.75%
D1-WT:136S/156T_well 4	3022	3723	81.17%	3184	3885	81.96%
D4-WT:P136S_well 1	1663	2086	79.72%			
D4-WT:P136S_well 2	4812	5237	91.88%			
D4-WT:P136S_well 3	6843	10519	65.05%			
D4-WT:P136S_well 4	2810	5914	47.51%			
D4-WT:A156T_well 1				3584	5708	62.79%
D4-WT:A156T_well 2				348	4088	8.51%
D4-WT:A156T_well 3				649	2277	28.50%
D4-WT:A156T_well 4				213	2004	10.63%
D4-WT:136S/156T_well 1	1745	4366	39.97%	1808	4453	40.60%
D4-WT:136S/156T_well 2	1757	2931	59.95%	1995	3166	63.01%
D4-WT:136S/156T_well 3	980	4285	22.87%	990	4375	22.63%
D4-WT:136S/156T_well 4	2246	5047	44.50%	2373	5101	46.52%

Supplementary Table 4: NGS summary of ferret samples

Sample (WT:136S/156T=1:1)	136S			156T		
	Variants Count	Coverage	Frequency	Variants Count	Coverage	Frequency
Ferret inoculum repeat 1	6988	18478	37.82%	7757	19372	40.04%
Ferret inoculum repeat 2	12658	25217	50.20%	13243	26033	50.87%
NW D1 Ferret 1	3582	15669	22.86%	4357	15755	27.66%
NW D1 Ferret 2	5073	13311	38.11%	4921	13613	36.15%
NW D1 Ferret 3	2301	12747	18.05%	2245	12605	17.81%
NW D3 Ferret 1	16	1414	1.13%	14	1459	0.96%
NW D3 Ferret 2 repeat 1	3	816*	0.37%	5	734*	0.68%
NW D3 Ferret 2 repeat 2	38	5211	0.73%	31	5367	0.58%
NW D3 Ferret 3	36	6627	0.54%	26	6372	0.41%
Lung D4 Ferret 1 repeat 1			no read			no read
Lung D4 Ferret 1 repeat 2			no read			no read
Trachea D4 Ferret 1 repeat 1	2	4473	<0.05	0	4690	<0.05
Trachea D4 Ferret 1 repeat 2	0	379*	<0.05	0	739*	<0.05
NT D4 Ferret 1 repeat 1	33	9824	0.34%	30	8782	0.34%
NT D4 Ferret 1 repeat 2	9	2201	0.41%	5	2230	0.22%
Lung D4 Ferret 2	1945	20095	9.63%	1758	18705	9.40%
Trachea D4 Ferret 2	88	19870	0.44%	87	18200	0.48%
NT D4 Ferret 2	53	7325	0.72%	39	6871	0.57%
Lung D4 Ferret 3	792	9230	8.58%	765	8807	8.69%
Trachea D4 Ferret 3	19	12738	0.15%	2	10448	0.02%
NT D4 Ferret 3	10	2306	0.43%	5	2112	0.24%

*: The data with lower coverage and threshold were read manually from reads tracks, which is not validated and provided for reference only. Frequency <1% is not validated and provided for reference only.

Supplementary Table 5: Alignment of H5 HA from Clade 2.3.4.4b, Genotype B3.13, US human cases

Annotation	H5 mature HA numbering	EPI3171488|HA|A/Texas/37/2024	EPI3556416|HA|A/Missouri/121/2024	EPI3334182|HA|A/Michigan/90/2024	EPI3621204|HA|A/California/155/2024	EPI3731132|HA|A/California/213/2024	EPI3674117|HA|A/California/181/2024	EPI3621212|HA|A/California/168/2024	EPI3726669|HA|A/California/195/2024	EPI3630030|HA|A/California/173/2024	EPI3591633|HA|A/California/135/2024	EPI3726682|HA|A/California/194/2024	EPI3641366|HA|A/California/150/2024	EPI3602471|HA|A/California/147/2024	EPI3467521|HA|A/Colorado/139/2024	EPI3783209|HA|A/California/216/2024	EPI3610171|HA|A/California/152/2024	EPI3467505|HA|A/Colorado/138/2024	EPI3610163|HA|A/California/153/2024	EPI3467513|HA|A/Colorado/137/2024	EPI3437006|HA|A/Colorado/109/2024	EPI3630018|HA|A/California/151/2024	EPI3674141|HA|A/California/191/2024	EPI3674149|HA|A/California/193/2024
	88	D			G	G	G	G	G	G	G	G	G	G		G	G		G			G	G	G
	131	V				M	M		M				M	M		M						M	M	M
Antigenic site A	136	P	S																					
Antigenic site B; S/T=Glycosylation	156	A	T																					
	234	K																					R	
	309	N	N/A																					
	310	K	N/A																					
	311	L	N/A																					
	312	V	N/A																					
	313	L	N/A																					
	314	A	N/A																					
	315	T	N/A																					
	316	G	N/A																					
	317	L	N/A																					
	318	R	N/A																					
	319	N	N/A																					
	320	S	N/A		N	N	N	N	N	N	N	N	N	N		N	N		N			N	N	N
	321	P	N/A																					
	322	L	N/A																					
	323	R	N/A																					
	324	E	N/A																					
	325	K	N/A																					
	326	R	N/A																					
	327	R	N/A																					
	328	K	N/A																					
	329	R	N/A																					
HA2	330	G	N/A																					
HA2	331	L	N/A																					
HA2	332	F	N/A																					
HA2	333	G	N/A																					
HA2	334	A	N/A																					
HA2	335	I/K	N/A	I	I	I	I	I	I	I	I	I	I	I	I	I	I	I	I	I	I	I	I	I
HA2	355	H															H/Q							
HA2	510	V																I		I				
HA2	554	Y		H																				

N/A: Sequence not available

Amino acid differences among the HA of A(H5N1) isolates from the 2.3.4.4b genetic clade. HA sequences were analyzed using ClustalW multiple alignment in BioEdit software. Different amino acids referred to the HA sequence of A/Texas/37/2024 virus were noted.

Supplementary Table 6: Alignment of N1 NA from Clade 2.3.4.4b, Genotype B3.13, US human cases

Annotation	N1 numbering	EPI3171488|NA|A/Texas/37/2024	EPI3556416|NA|A/Missouri/121/2024	EPI3334182|NA|A/Michigan/90/2024	EPI3621204|NA|A/California/155/2024	EPI3731132|NA|A/California/213/2024	EPI3674117|NA|A/California/181/2024	EPI3621212|NA|A/California/168/2024	EPI3726669|NA|A/California/195/2024	EPI3630030|NA|A/California/173/2024	EPI3591633|NA|A/California/135/2024	EPI3726682|NA|A/California/194/2024	EPI3641366|NA|A/California/150/2024	EPI3602471|NA|A/California/147/2024	EPI3467521|NA|A/Colorado/139/2024	EPI3783209|NA|A/California/216/2024	EPI3610171|NA|A/California/152/2024	EPI3467505|NA|A/Colorado/138/2024	EPI3610163|NA|A/California/153/2024	EPI3467513|NA|A/Colorado/137/2024	EPI3437006|NA|A/Colorado/109/2024	EPI3630018|NA|A/California/151/2024	EPI3674141|NA|A/California/191/2024	EPI3674149|NA|A/California/193/2024
	5	Q																R		R				
	30	I	T																					
	51	Q		R																				
	71	N			S	S	S	S	S	S	S	S	S	S	S	S	S	S	S	S	S	S	S	S
	84	T						A																
	122	I			V																			
	269	M								V														
	286	G	N/A																					
	287	D	N/A																					
	288	I	N/A																					
	289	M	N/A																					
	290	C	N/A																					
	291	V	N/A																					
	292	C	N/A																					
	293	R	N/A																					
	294	D	N/A																					
	295	N	N/A																					
	296	W	N/A																					
	297	H	N/A																					
	298	G	N/A																					
	299	S	N/A																					
	300	N	N/A																					
	301	R	N/A																					
	302	P	N/A																					
	303	W	N/A																					
	304	V	N/A																					
	305	S	N/A																					
	306	F	N/A																					
	307	N	N/A																					
	308	Q	N/A																					
	309	N	N/A																					
	310	L	N/A																					
	311	E	N/A																					
	312	Y	N/A																					
	313	Q	N/A																					
	314	I	N/A																					
	315	G	N/A																					
	316	Y	N/A																					
	317	I	N/A																					
	318	C	N/A																					
	319	S	N/A																					
	320	G	N/A																					
	321	I	N/A																					
	322	F	N/A																					
	323	G	N/A																					
	324	D	N/A																					
	325	N	N/A																					
	326	P	N/A																					
	327	R	N/A																					
Antigenic site I	328	P	N/A																					
Antigenic site I	329	N	N/A																					
Antigenic site I	330	D	N/A																					
Antigenic site I	331	G	N/A																					
Antigenic site I	332	T	N/A																					
Antigenic site I	333	G	N/A																					
Antigenic site I	334	S	N/A																					
	335	C	N/A																					
Antigenic site II	336	S	N/A																					
Antigenic site II	337	P	N/A																					
Antigenic site II	338	M	N/A																					

Antigenic site II	339	P	N/A																					
Antigenic site II	340	S	N/A																					
Antigenic site II	341	N	N/A																					
Antigenic site II	342	G	N/A																					
Antigenic site II	343	A	N/A																					
Antigenic site II	344	Y	N/A																					
	345	G	N/A																					
	346	V	N/A																					
	347	K	N/A																					
	348	G	N/A																					
	349	F	N/A																					
	350	S	N/A																					
	351	F	N/A																					
	352	K	N/A																					
	353	Y	N/A																					
	354	G	N/A																					
	355	N	N/A																					
	356	G	N/A																					
	357	V	N/A																					
	358	W	N/A																					
	359	I	N/A																					
	360	G	N/A																					
	361	R	N/A																					
	362	T	N/A																					
	363	K	N/A																					
Antigenic site III	364	S	N/A																					
Antigenic site III	365	T	N/A																					
Antigenic site III	366	S	N/A																					
Antigenic site III	367	S	N/A																					
	368	R	N/A																					
	369	S	N/A										N											
	370	G	N/A																					
	371	F	N/A																					
	372	E	N/A																					
	373	M	N/A																					
	374	I	N/A																					
	375	W	N/A																					
	376	D	N/A																					
	377	P	N/A																					
	378	N	N/A																					
	379	G	N/A																					
	380	W	N/A																					
	381	T	N/A																					
	382	E	N/A																					
	383	T	N/A																					
	384	D	N/A																					
	385	S	N/A																					
	386	S	N/A																					
	387	F	N/A																					
	388	S	N/A																					
	389	V	N/A																					
	390	K	N/A																					
	391	Q	N/A																					
	392	D	N/A																					
	393	I	N/A																					
	394	V	N/A																					
	395	E	N/A																					
Antigenic site IV	396	I	N/A																					
Antigenic site IV	397	T	N/A																					
Antigenic site IV	398	D	N/A																					
Antigenic site IV	399	W	N/A																					
	400	S	N/A																					
	401	G	N/A																					
	402	Y	N/A																					
	403	S	N/A																					
	404	G	N/A																					
	407	V	F																					
	436	I												I/M										
	439	S									G													
	475	T				N																		

N/A: sequence not available

Amino acid differences among the N1 NA of the A(H5N1) isolates from the 2. 3. 4. 4b genetic clade. NA sequences were analyzed using ClustalW multiple alignment in BioEdit software. Putative NA antigenic sites (I to VII) were annotated. Different amino acids referred to the NA sequence of A/Texas/37/2024 H5N1 virus were presented.
